# Radiation-free flexible ureteroscopy for kidney stone treatment

**DOI:** 10.1080/2090598X.2019.1606381

**Published:** 2019-04-24

**Authors:** Braulio O. Manzo, Edgard Lozada, Gildardo Manzo, Héctor M. Sánchez, Francisco Gomez, Alejandro Figueroa, Adrian Gonzalez

**Affiliations:** Department of Urology, Hospital Regional de Alta Especialidad del Bajío, Leon, Mexico

**Keywords:** Ureteroscopy, renal stones, laser, lithotripsy, fluoroscopy, radiation

## Abstract

**Objectives**: To evaluate the safety and effectiveness of flexible ureterorenoscopy (fURS) with holmium laser lithotripsy for treating kidney stones without fluoroscopy as method of best practice for patients and endourologists.

**Patients and Methods**: All patients treated for kidney stones by fURS with holmium laser lithotripsy from February 2016 to February 2017 were retrospectively evaluated. The patients’ demographic characteristics, stone features (size, number, and location), surgical variables (use of fluoroscopy, operative and fluoroscopy time), complications, and success rate (employing stone-free rate [SFR]), were included in the analysis.

**Results**: In all, 100 patients met the inclusion criteria: 33 fURS were performed under fluoroscopy (Group 1) and 67 without it (Group 2). The mean operating time was 94.33 vs 98.29 min (*P* = 0.888), respectively. The mean stone volume was 78.5 vs 82.4 mL (*P* = 0.885), respectively. The SFR was 63.6% and 64.2% (*P* = 0.771), the perioperative complications rate was 18.2% vs 11.9% (*P* = 0.285), and the postoperative complications rate was 24.2% and 10.4%, in groups 1 and 2 respectively (*P* = 0.174).

**Conclusions**: fURS with holmium laser lithotripsy without fluoroscopy was a feasible and safe treatment for kidney stones. There was no difference between the use of fluoroscopy or not regarding complications or SFR. Thus, we can reduce the risks of radiation exposure to patients and medical staff whilst maintaining surgical success. However, multicentre randomised controlled studies are necessary to evaluate fluoroless URS further and to confirm our present results.

**Abbreviations:** PTFE: polytetrafluoroethylene; SFR: stone-free rate; (f)URS: (flexible) ureterorenoscopy/ureterorenoscopies; US: ultrasonography

## Introduction

In recent years, the incidence of urinary stones has increased around the world. This rise has led to an increase of 83% in the number of flexible ureterorenoscopies (fURS) performed in the USA between 1994 and 2004. Additionally, the augmented use of fURS for treating kidney stones has been facilitated by the improvements in the new flexible ureteroscopes, which allow easier access to the urinary tract and the collecting system, with excellent visualisation [1,2].

Since the origin of fURS, urologists have included fluoroscopy at the time of the procedure, and since then, fluoroscopy has played a crucial role in this specific procedure. However, the USA Food and Drug Administration (FDA) has been concerned about fluoroscopy radiation exposure of patients and surgeons and recently has recommended reducing fluoroscopy as much as possible in all surgical procedures as a best practice.

Over the years, the harmful effects of radiation on the human body have been demonstrated, putting endourologists at a higher risk due to the radiation absorbed during their working careers [3,4]. That is why recently there has been a lot of effort to reduce the use of fluoroscopy in endourological procedures. Many authors have published various techniques and protocols for reducing radiation exposure as much as possible in endourological stone treatments. All of these previous studies still used fluoroscopy at least in one step of the surgical procedure, still exposing the patients with stones and surgeons to some radiation, contributing to increasing the allowed annual dose of radiation. Even with the vast amount of studies about low radiation protocols, to our knowledge, this is the first study to evaluate a protocol of fURS completely free of radiation for the treatment of renal stones [5–20].

## Patients and methods

We included all patients with renal stones of <15 mm, who were submitted to our protocol of fluoroless-fURS with holmium laser lithotripsy (see fluoroless surgical technique) from February 2016 to February 2017. All the patients that met the inclusion criteria were retrospectively evaluated and compared to a subset of patients previously treated with our fluoroscopy protocol. Two groups were created for statistical analysis, Group 1: fluoroscopy and Group 2: no fluoroscopy. All the surgical procedures reviewed were performed by a single experienced urologist that performs >100 fURS/year. The fluoroscopy group (Group 1) included those patients treated before the surgeon began the fluoroless protocol for fURS.

Patients’ demographic characteristics, stone features (size, number, and location), fluoroscopy use, operative time (in minutes) and fluoroscopy time (in seconds), presence of hydronephrosis, perioperative and postoperative complications, as well as the stone-free rate (SFR), were all retrospectively evaluated and compared between both groups. The diagnosis was made by non-contrast CT, and the assessment of the SFR was made using the same method 3 months after the fURS, as the hospital’s protocol stated. Residual stones were defined as renal stones of >2 mm encountered at postoperative CT. All patients who did not have complete data for the retrospective analysis, those with ipsilateral or contralateral ureteric stones, and those with additional ipsilateral or contralateral simultaneous endourological surgical procedures such as percutaneous nephrolithotomy, semi-rigid URS, ureteric balloon dilatation, laser endopyelotomy, and open or laparoscopic nephrolithotomy, were excluded from the analysis.

The commercial Statistical Package for the Social Sciences (SPSS®) program for Windows 10 was used for statistical analysis (SPSS Inc., IBM Corp., Armonk, NY, USA). Descriptive statistics were used to determine the variables’ distribution. Continuous variables are reported as means and standard deviations (SDs), and to compare the results between the two groups a Student’s *t*-test was used for independent groups. The presence of complications and demographic variables were expressed as frequencies and percentages. Comparison of the results was made using chi-squared or Fisher’s exact tests depending on each case. In the cases that did not meet assumptions of normality for analysis, nonparametric statistics were used and the Mann–Whitney test used. A *P* < 0.05 was considered to indicate statistical significance.

### Fluoroless surgical technique

After a detailed cystoscopy, a hybrid 0.089 cm (0.035 inch) guidewire was introduced (Sensor™; Boston Scientific, Marlborough, MA, USA) into the ureteric meatus (the insertion of the guidewire was halted in the presence of resistance). Then, a ureteric access sheath was advanced over the hybrid guidewire, and the insertion process was terminated at the slightest resistance, with the purpose of avoiding any ureteric or renal damage. Adequate ureteric access sheath position was verified by direct endoscopic vision with the flexible ureteroscope. In those cases in which we saw that the ureteric access sheath could not reach the PUJ but could go through the uretero-vesical junction, the ureteric access sheath was left in place, and fURS was completed. If the introduction of the ureteric access sheath failed, then we advanced the flexible ureteroscope through the same hybrid guidewire and performed ureteronephroscopy.

After the stones were localised by direct endoscopic vision (all lower pole calyx stones were re-positioned to an upper or medial pole calyx whenever possible and for those in which re-positioning was not possible *in situ* lithotripsy was performed), lithotripsy was performed with a holmium laser in each case (laser energy parameters were dependent upon stone hardness and size). All visible fragments >2 mm were extracted with a nitinol basket in all patients, and a complete fURS was accomplished at the end of the procedure in each case to evaluate every single calyx, the renal pelvis, and the ureter. Once we corroborated the endoscopic stone-free status, the flexible ureteroscope was extracted under direct vision evaluating the ureter thoroughly. In those patients with a ureteric wall lesion classified as grade 2–3, we left a double-pigtail stent at the end of the procedure. For co-location of the double-pigtail stent, a hybrid guidewire was positioned through the flexible ureteroscope in the upper or middle pole calyx. Afterwards, we proceeded to extract the flexible ureteroscope over the guidewire left in the desired calyx. Finally, by cystoscopy, we introduced the double-pigtail stent through the guidewire under direct endoscopic vision with the cystoscope until we could see that the distal curl of the stent had taken its shape adequately. Finally, we corroborated the proximal curl location with renal ultrasonography (US).

Postoperative complications were classified according to the Clavien–Dindo classification. The fluoroscopic machine was always available for use in the surgical room in all cases.

## Results

Over a 1-year period, 100 patients met the inclusion criteria and were included in our statistical analysis. In the fluoroscopy group, there were 33 patients (Group 1) and in the fluoroless group 67 patients (Group 2).

The patients’ demographic characteristics showed that both groups were homogeneous without any significant statistical difference between them (Table 1). The average patients’ age was 49 years for both groups. Calculi features are also summarised in Table 1.

**Table 1.** Demographic data and stone features.

Variable	Group 1 Fluoroscopy	Group 2 Without fluoroscopy	*P*
Gender, *n* (%)			0.017*
Female	8 (24.2)	33 (49.3)	
Male	25 (75.8)	34 (50.7)	
Age, years, mean (SD)	48.75 (14.56)	49.16 (12.58)	0.367**
Stone number, *n* (%)			0.543***
1	18 (54.5)	40 (59.7)	
2	7 (21.2)	15 (22.4)	
3	4 (12.1)	6 (9)	
4	2 (6.1)	0	
5	1 (3)	2 (3)	
6	0	1 (1.5)	
Maximum stone diameter, mm, median (IQR)	13 (8.5–1.6)	10 (8–19.5)	0.671****
Stone volume, mL, median (IQR)	78.5 (50–137.4)	82.4 (50–166.4)	0.885****
Previous PCNL, *n* (%)	10 (30.3)	18 (26.8)	0.719*
Previous SWL, *n* (%)	10 (30.3)	18 (26.9)	0.814*
Double-pigtail stent, *n* (%).	12 (36.4)	31 (41.6)	0.47***

PCNL, percutaneous nephrolithotomy; SWL, shockwave lithotripsy. *Comparison between groups was performed using the chi-squared test and results are reported as frequency and percentage. **Results are reported as a mean and standard deviation. For comparison between independent groups Student’s *t*-test was used. ***Comparison between groups was performed using Fisher´s exact test. Results are reported as frequency and percentage. ****Comparison between groups was performed using the Mann–Whitney *U*-test. Results are reported as median and interquartile range (IQR).

The perioperative variables that were evaluated are shown in Table 2. In all the patients in which fluoroscopy was used, the mean radiation time was 8 s. The mean operative time was 94.33 min in Group 1 and 98.29 min in Group 2, with no statistical difference between them (*P* = 0.88). A ureteric access sheath was used in 26 patients in Group 1 and 65 patients in Group 2 (*P* = 0.002).

**Table 2.** Intraoperative variables.

Variable	Group 1 Fluoroscopy	Group 2 Without fluoroscopy	*P*
Fluoroscopy time, s, mean (range)	8 (3.5–11.5)		
Operative time, min, mean (SD)	94.33 (37.2)	98.29 (49.4)	0.888b
Ureteric access sheath, *n* (%)	26 (78.8)	65 (97.1)	0.002a
10/12 F	0	10 (14.9)	0.028a
11/13 F	23 (69.7)	55 (82)	0.201a
12/14 F	3 (9.09)	0	0.033a

aComparison between groups was performed using Fisher´s exact test. Results are reported as frequency and percentage.

bResults are reported as a mean (SD). For comparison between independent groups the Student’s *t*-test was used.

There were eight and seven perioperative complications in each group, respectively (*P* = 0.285), with no statistically significant difference (Table 3). Based on the Clavien–Dindo classification there were no grade IV or V complications in any group (the specific complication grades per group are summarised in Table 3).

**Table 3.** Perioperative and postoperative complications.

Variable	Group 1 Fluoroscopy *N* = 33	Group 2 Without fluoroscopy *N* = 67	*P*
Postoperative complications, *n* (%)	8 (24.2)	7 (10.4)	0.174a
Clavien–Dindo, *n*	Grade I Total 2	Grade I Total 3	
	Pain 2	Nausea 1	
		Haematuria 1	
		Pain 1	
	Grade II Total 2	Grade II Total 0	
	Urinary sepsis 1		
	Pneumonia 1		
	Grade IIIa Total 4	Grade IIIa Total 4	
	Urethral stenosis 3	Urethral stenosis 3	
	Ureteric stenosis 1	Ureteral stenosis 1	
Perioperative complications, *n* (%)	6 (18.2)	8 (11.9)	0.285a
Clavien–Dindo, *n*	Grade I Total 3	Grade I Total 4	
	Haemorrhage 1	Haemorrhage 3	
	Urethral false passage 2	Ureteral mucosal laceration 1	
	Grade II Total 1	Grade II Total 0	
	Bacteraemia 1		
	Grade IIIa Total 2	Grade IIIa Total 4	
	Incomplete lithotripsy 2	Incomplete lithotripsy 4	
Fever, *n* (%)	3 (9.1)	4 (6)	0.265b
Days of hospital stay, *n* (%)			0.885a
1	29 (87.9)	63 (86.6)	
2	3 (9.1)	1 (1.5)	
3	0	1 (1.5)	
4	1 (3)	0	
5	0	2 (3)	

aComparison between groups was performed using the chi-squared test and results are reported as frequency and percentage.

bComparison between groups was performed using the Fisher’s exact test. Results are reported as frequency and percentage.

For postoperative complications (0–3 months), there was no statistically significant difference (*P* = 0.174) between both groups, and similarly, there were no grade IV or V complications based on the Clavien–Dindo classification (Table 3).

Postoperative fever was present in three (9.1%) patients in Group 1 and four (6%) in Group 2 (*P* = 0.265). In relation to the hospital stay, 87.9% and 86.6% of the patients required only 1 day of hospital stay, respectively (*P* = 0.885).

Group 1 had a SFR of 63.6%, whilst in Group 2 it was 64.2%, with no statistically significant difference between the groups (*P* = 0.771).

## Discussion

The international urological community is beginning to use protocols to lower the use of fluoroscopy and replace it with methods that do not emit radiation (e.g. use of US) for all the endourological procedures to treat renal stones [2,5–16]. This international trend also applies to paediatric patients [17].

Greene et al. [1] reported no difference in primary outcomes (the SFR, surgical time and complications) when comparing their protocol of reduced fluoroscopy with their standard protocol. They concluded that the fluoroscopy time could be reduced by 82% without altering the primary outcomes. Although in their study they do not refer to whether URS was either rigid or fURS and neither gave details about the location(s) of the stones treated.

Tepeler et al. [5], 1 year later (2012), performed a retrospective review of 93 patients who were treated by semi-rigid URS without fluoroscopy just for ureteric stones. Similar to our surgical protocol, the main steps, such as the introduction of the guidewires, ureteric stents, and dilatation balloons were guided by tactile signals or visual cues, reinforcing the idea that, it is possible to perform a URS without radiation. They reported a failure to complete the procedure successfully in 7.6% of the patients in whom fluoroscopy was not used. Unlike Tepeler et al. [5], our surgical procedure was completed successfully in all the patients in the fluoroless group with no need for fluoroscopy use. It is important to say that we did not include patients that needed balloon dilatation and we did not perform balloon dilatation before fURS.

Hsi and Harper [8] in their prospective evaluation of 162 semi-rigid URS and fURS, stated that 75% of the patients required no fluoroscopy during the surgery, but they did at the final step of the surgical procedure for ureteric stent placement in each case. So their surgical procedure is still reliant on fluoroscopy radiation. In our protocol, stent placement was done after we left a hybrid guidewire in the desired renal cavity under direct endoscopic vision. We verified the final position of the double-pigtail stent when we saw the proper formation of the distal curl by the cystoscope and with renal US for the proximal curl.

Olgin et al. [7] published a completely fluoroless surgical technique. As an essential key to omit fluoroscopy radiation in their surgical protocol, they used a hydrophilic guide wire (Ter-UMO Medical Corporation, Irvine, CA) as an access guidewire, with the purpose of avoiding ureteric trauma, but this guidewire had to be changed to a conventional polytetrafluoroethylene (PTFE) guidewire (working guide wire). This process could mean a necessity to use and interchange at least two different guidewires during the surgical procedure, which could result in more ureteric manipulation and the requirement of additional medical material. They concluded that URS could be performed safely without the use of radiation, except for those patients with ureteric stenosis, calcified catheters, anatomical abnormalities or impacted urinary tract calculi.

Clayman et al. [19] compared different guidewires used in urology and concluded that hydrophilic guides with a soft tip, made of nitinol, are the best ones for ureteric access, whilst more rigid ones like those wholly made of PTFE work best for coaxial passage of catheters, stents and sheaths.

As stated by Cayman et al. [19], in our surgical protocol the key for avoiding fluoroscopy, besides the essential gentile manoeuvres, is the use of a hybrid guidewire, which with its soft hydrophilic tip allows the surgeon to use it as an initial access guide. Its soft hydrophilic tip diminishes the risk of a ureteric perforation or a false passage, but we emphasise that a ureteric lesion can be possible even with a hybrid guidewire. So the guidewire should be gently introduced and if there is any resistance then the guidewire introduction has to be stopped. It is important to say that when we found any resistance at guidewire introduction, we left it in place and we performed a URS to see the place where the guidewire was positioned, and then we relocated the guidewire if it was necessary.

Concerning our present outcomes, the SFR (defined as no fragments of >2 mm on non-contrast CT evaluated at 3-months postoperatively) was similar between the two groups (63.6% vs 64.2%), which suggests that the effectiveness of URS for treating kidney stones does not decrease without fluoroscopy. Comparing the SFR, with those reported by other authors we had a lower success rate. However, we may be able to explain our lower success rate because we evaluated the residual fragments by CT. CT has been shown to have a higher sensitivity and specificity for residual stones detection than US or radiography, as CT can detect smaller and low-density stones.

It is noteworthy that the only series of patients comparing complete fluoroless-fURS with a fluoroscopy cohort had a SFR of 92% for both groups. However, they do not refer which method they used for the SFR evaluation [7].

For postoperative complications, the presence of fever or the hospital stay, we did not find a statistically significant difference between both groups, showing that avoiding the use of fluoroscopy during the surgical procedure does not increase the surgical risk or the complications.

All patients diagnosed with renal stones are subjected to multiple diagnostic and therapeutic procedures (tomography, excretory urography, plain abdominal radiography, pyelography, and fluoroscopy) involving a cumulative large radiation load. Moreover, endourologists and urologists dedicated mainly to urolithiasis treatment, have significant exposure to the deleterious effects of radiation in their working life. Therefore, we believe it is essential, as a best practice, to avoid or to reduce to a minimum as much as possible the use of fluoroscopy during fURS for the kidney stones treatment.

Now we have abandoned the routine use of fluoroscopy for fURS in the treatment of renal stones, although a C-arm is always available for its use in the operative room. Additionally, we have implemented an education programme to teach our fellows and residents in this surgical protocol in a systematic manner.

The ureteric access sheath introduction is a critical step and requires a keen sense of touch. Even with fluoroscopy, this sensitive control is necessary to know if the ureteric access sheath has a free passage through the ureter to avoid ureteric damage. This sense of touch is acquired with experience, so a novice surgeon needs previous experience in fURS or to be in a tutored programme to gradually learn the way to perform fURS without fluoroscopy, including the guidewire and ureteric access sheath introduction.

We still use fluoroscopy for those patients that have an abnormal collecting system, e.g. horseshoe kidney, complete or incomplete duplicated system, previous renal open surgery or transplanted kidneys.

We have passed from using fluoroscopy to avoiding it, and for those urologists that still use fluoroscopy in fURS and want to reduce it; we recommend the gradual diminution in fluoroscopy use until they get the expertise and feel comfortable without it.

Additionally, we still use fluoroscopy for ureteric endoscopic dilatation with a balloon and a programmed laser endopyelotomy, and we want to emphasise that we do not perform fURS and balloon dilatation simultaneously.

We recognise the retrospective character of our present study; even so, we believe our results can be seen as a justification for a prospective and randomised controlled trial that can test the effectiveness and the safety of complete fluoroless-URS for the treatment of kidney stones.

We found a significantly higher proportion of men than women in the fluoroscopy group (Group 1), but in the fluoroless group (Group 2) we had a homogeneous population. This difference in gender between groups is inherent to the retrospective design of our study. This issue could be seen as a weakness of our present study and could be resolved with a prospective and randomised controlled study.

As a strength of our present study, we emphasise that the evaluation of residual stones was performed by non-contrast CT in every single patient, so we can be sure about the real SFRs between both groups, and importantly there was no significant difference between the SFRs.

## Conclusions

Based on our present results, we can conclude that performing URS with holmium laser lithotripsy without fluoroscopy is feasible and safe for most patients with kidney stones. It can reduce the patients’ and surgeons’ risks from radiation exposure without increasing the surgical risk or complications for the patients, whilst maintaining the success of the surgery. However, we recommend this fluoroless protocol be used in high-volume centres where urologists have enough experience in fURS, and we still suggest the use of fluoroscopy for low-volume centres and complicated cases. Finally, for low-volume centres, we recommend trying to gradually reduce fluoroscopy use until the surgical skills, confidence, and experience of the surgeon(s) is enhanced, and not to attempt this approach without sufficient experience. The patients’ safety is always paramount and should never be at risk. We present our protocol as a way to reduce radiation safely, but we recommend fluoroscopy use whenever it is needed.

Multicentre randomised controlled trials are needed to evaluate fluoroless-URS further and confirm our present results.
